# 784 A Burn Resting Hand Orthosis Fit Checklist

**DOI:** 10.1093/jbcr/irac012.335

**Published:** 2022-03-23

**Authors:** Renee Warthman, Andria Martinez, Derek Murray, Karen Richey, Kevin N Foster

**Affiliations:** Arizona Burn Center Valleywise Health, Phoenix, Arizona; Arizona Burn Center Valleywise Health, Phoenix, Arizona; Arizona Burn Center Valleywise Health, Phoenix, Arizona; Arizona Burn Center Valleywise Health, Phoenix, Arizona; The Arizona Burn Center Valleywise Health, Phoenix, Arizona

## Abstract

**Introduction:**

Resting hand orthoses (RHO) are often used to manage hand burns but can be difficult for inexperienced clinicians to fabricate, assess, and utilize without causing complications to the patient. Previously we had shared data on how we reduced RHO related complications by utilizing burn and hand certified staff to fabricate RHOs.

**Methods:**

Advanced clinicians certified in either hand, burn, or both were queried to describe the methodology they utilized to fabricate an effective RHO. From this query, a list of conditions was created. These conditions were presented to the entire group of certified clinicians and re-examined. Conditions were recorded if consensus was reached on their inclusion for a guide to fabricate a successful RHO.

**Results:**

A 14-condition checklist, with 8 essential items, was developed. It is intended that this checklist can be utilized by less experienced clinicians to guide safe and effective RHO management.

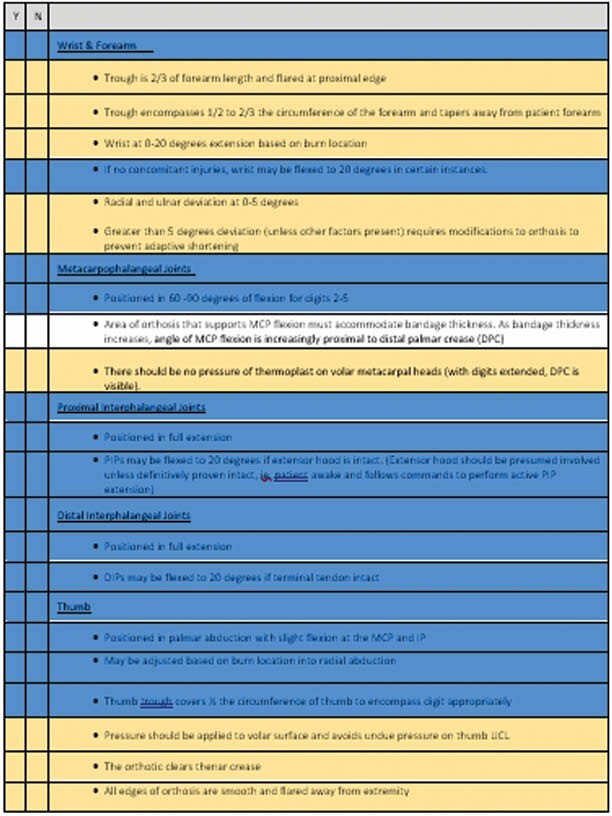

**Conclusions:**

The checklist is a straightforward way to assess commonly identified issues with RHO and offers a potential resource to manage effective fabrication for novice or inexperienced clinicians.

